# Gadolinium Complexes as Contrast Agent for Cellular NMR Spectroscopy

**DOI:** 10.3390/ijms21114042

**Published:** 2020-06-05

**Authors:** Nat Sakol, Ayako Egawa, Toshimichi Fujiwara

**Affiliations:** Institute for Protein Research, Osaka University, 3-2 Yamadaoka, Suita, Osaka 565-0871, Japan; chalermponk@protein.osaka-u.ac.jp (N.S.); e-ayako@protein.osaka-u.ac.jp (A.E.)

**Keywords:** Gd-DOTA, GdCl_3_, paramagnetic relaxation enhancement, solid-state NMR, in-cell NMR

## Abstract

Aqua Gd^3+^ and Gd-DOTA (gadolinium-1,4,7,10-tetraazacyclododecane-1,4,7,10-tetraacete) complexes were studied as a contrast agent in cellular NMR (nuclear magnetic resonance) spectroscopy for distinguishing between intracellular and extracellular spaces. The contrast agents for this purpose should provide strong paramagnetic relaxation enhancement and localize in the extracellular space without disturbing biological functions. Cell membrane permeability to Gd complexes was evaluated from the concentrations of gadolinium complexes in the inside and outside of *E. coli* cells measured by the ^1^H-NMR relaxation. The site-specific binding of the complexes to *E. coli* cells was also analyzed by high-resolution solid-state ^13^C-NMR. The aqua Gd^3+^ complex did not enhance *T*_1_ relaxation in proportion to the amount of added Gd^3+^. This Gd^3+^ concentration dependence and the ^13^C-NMR indicated that its strong cytotoxicity should be due to the binding of the paramagnetic ions to cellular components especially at the lipid membranes. In contrast, Gd-DOTA stayed in the solution states and enhanced relaxation in proportion to the added amount. This agent exhibited strong *T*_1_ contrast between the intra- and extracellular spaces by a factor of ten at high concentrations under which the cells were viable over a long experimental time of days. These properties make Gd-DOTA suitable for selectively contrasting the living cellular space in NMR spectroscopy primarily owing to its weak interaction with cellular components.

## 1. Introduction

Paramagnetic relaxation enhancement (PRE) is the acceleration of nuclear spin relaxation by magnetic interactions with unpaired electron spins. Since the magnetic interaction depends on the distance between nuclear and electron spins, this effect provides the structural information for proteins and intermolecular interactions [1,2,3,4]. For instance, measurement of PRE is one standard method in NMR (nuclear magnetic resonance) spectroscopy for probing the location of peptides embedded in lipid bilayer membranes [5,6,7,8]. The lanthanide metal ion Gd^3+^ has the largest number of unpaired electrons in all transition metal ions, so it should provide the strongest PRE in biological systems. Because of its isotropic *g* tensor, Gd^3+^ ions do not alter the resonance frequencies by pseudo contact shift. The PRE effect of the Gd^3+^ ion is commonly utilized in MRI as a contrast agent for clinical analysis [9,10,11,12]. The Gd^3+^ ions used in MRI have to be sealed as a stable complex [12,13], otherwise Gd^3+^ could strongly influence biological processes. For example, since the radius of Gd^3+^ is close to that of Ca^2+^, Gd^3+^ disturbs the functions of Ca^2+^ binding enzymes [14,15,16,17]. The gadolinium-1,4,7,10-tetraazacyclododecane-1,4,7,10-tetraacete complex, Gd-DOTA, is one of Gd chelate complexes used clinically because of its high stability in the environment of human organs [17,18]. Although some gadolinium complexes including Gd-DOTA complex have been proven to be safe for humans, it has been reported that these complexes could be internalized into human cells [19,20,21,22,23].

Emerging cellular NMR spectroscopy draws much attention in structural biology because it elucidates the structure and function of macromolecules directly in native cellular environments [24,25,26,27,28]. Nevertheless, several informative NMR spectroscopic methods are still not applied to cellular NMR. For example, there is still no information on the application of PRE to distance measurements in cells, while it is often applied to NMR spectroscopy of biological macromolecules [1,29,30]. To make use of PRE in NMR for obtaining cellular and biomolecular information, the paramagnetic agent has to be harmless and its distribution in the cellular sample should be known for the analysis. The Gd-DOTA complex is one candidate paramagnetic agent owing to its low toxicity. In this study, we employed aqua Gd^3+^ complex, i.e., [Gd(H_2_O)_8_]^3+^, and Gd-DOTA complexes as paramagnetic agents to locate molecules in *Escherichia coli* cells which were selected as a model cell system [31]. We measured the distribution of Gd complexes in the *E. coli* cellular solution from the longitudinal relaxation process of water proton magnetization and evaluated the permeability of the cell membranes to these complexes. We also verified the different interactions of Gd-DOTA complex and aqua Gd^3+^ complex with cellular components in living *E. coli* cells by high-resolution solid-state ^13^C-NMR spectroscopy, and examined the cytotoxicity of these complexes quantitatively.

## 2. Results

### 2.1. NMR Spectra of E. coli Sample in Absense of Gadolinium Complex

**Figure 1:**
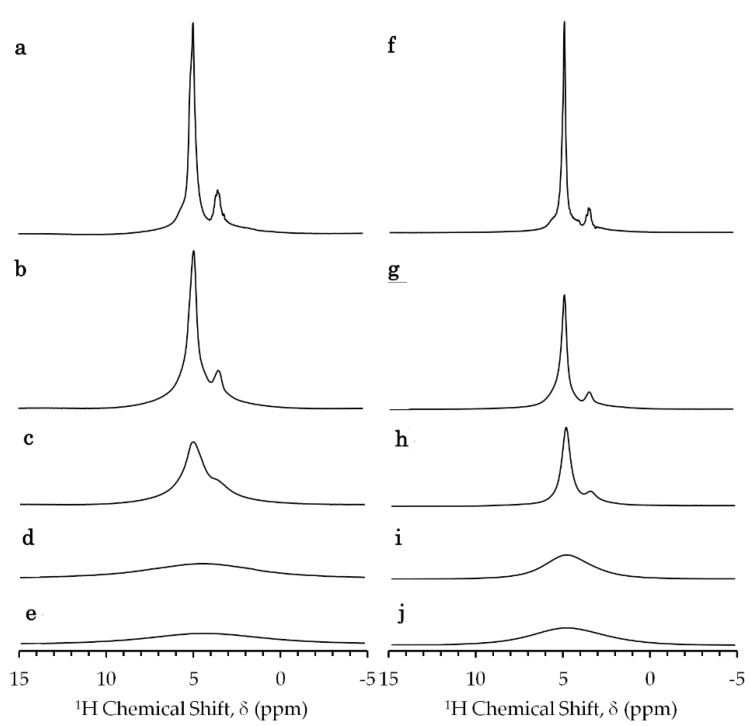
^1^H-NMR spectra of *E. coli* sample mixed with (**a**–**e**) aqua Gd^3+^ and (**f**–**j**) Gd-DOTA (gadolinium-1,4,7,10-tetraazacyclododecane-1,4,7,10-tetraacete complex) obtained by a single pulse Fourier transformation method at a ^1^H resonance frequency of 700 MHz and temperature of 273 K. Samples of 10 μL of (**a**) 0, (**b**) 12, (**c**) 25, (**d**) 149 and (**e**) 250 mM aqua Gd^3+^ solutions and 10 μL of (**f**) 0, (**g**) 15, (**h**) 25, (**i**) 150, and (**j**) 250 mM Gd-DOTA solutions were added to 40 μL of glycerol-*E. coli*. A constant receiver gain and a constant scaling factor for the Y-axis were used.

The ^1^H NMR spectra of glycerol-*E. coli* mixtures showed a strong peak of the hydroxyl proton of water and glycerol (Figure 1) as water accounts for about 70% of the sample (Supplementary Materials S1) [32]. The sample preparation of glycerol-*E. coli* mixtures is given in Section 4.3. The exchange rate of the water proton with the hydroxyl proton of glycerol was much faster than the relaxation rate 1/*T*_1_ of this peak. The smaller peak next to the hydroxyl peak was interpreted as the methine and methylene protons of glycerol. The peaks of the other cellular components did not appear significant because of the large chemical shift dispersion of those components and the lower concentrations in comparison to water and glycerol. The strong ^1^H–^1^H dipolar interactions in the large rigid cellular components should cause their resonances to be broadened. The reduction in signal height at higher Gd concentrations should be due to paramagnetic relaxation and dielectric loss.

The proton *T*_1_ relaxation components with the rate of 0.887 **±** 0.035 and 1.51 **±** 0.31 s^−1^ were assigned to the extracellular and intracellular water, respectively, for the reasons as follows. The intracellular *T*_1_ was calculated on the basis of the cellular water model by Persson and Halle [33,34]. In this model, the correlation time (*τ*_c_) of the first water hydration layer surrounding the cellular macromolecules is 15 times longer than *τ*_c_ of the bulk water. The intracellular water other than the first layer should have the same τ_c_ as the bulk water. The experimental intracellular *T*_1_ relaxation rate revealed *τ*_c_ of 5.36 and 83.6 ps with the viscosity η of 1.76 and 27.4 mPa.s for the bulk and first water hydration layer, respectively, as detailed in Supplementary Materials S2 and S3. The obtained τ_c_ of the intracellular bulk water, 5.36 ps, agrees with τ_c_ predicted from empirical parameters for the bulk water, 5.26 ps (Supplementary Materials S3). This agreement supports the assignment of the relaxation component with *R*_1_ = 1/*T*_1_ of 1.51 s^−1^ to the intracellular water. Figure S1 also supports the analysis.

To approximate the concentration of extracellular glycerol from *R*_1_, we measured *R*_1_ of water proton in a reference glycerol solution of 20% *v/v*. The experimental *R*_1_ of this glycerol solution of 0.98 s^−1^ was converted to η of 3.09 mPa.s and *τ*_c_ of 9.43 ps. The *R*_1_ of the extracellular water of the sample gave η of 2.64 mPa.s and *τ*_c_ of 8.06 ps. The extracellular water η of the sample relative to η of the reference glycerol solution indicated the extracellular glycerol concentration of about 18%. This concentration agrees with the concentration of glycerol in the extracellular solution of 16.9–22.7% *v*/*v* obtained from the added amount of glycerol under the assumption that the glycerol did not penetrate into the cytoplasm (Supplementary Materials S3). This agreement also supports the idea that the relaxation component with *R*_1_ of 0.887 s^−1^ should be assigned to the extracellular water.

### 2.2. PRE for Gadolinium Solutions

The *E. coli* sample in the presence of aqua Gd^3+^ and Gd-DOTA solutions provided similar ^1^H-NMR spectra (Figure 1). The methine and methylene peaks broadened and disappeared due to the transverse PRE with the increase in the gadolinium concentration of the solutions which were prepared by the procedure given in Section 4.3.

**Figure 2:**
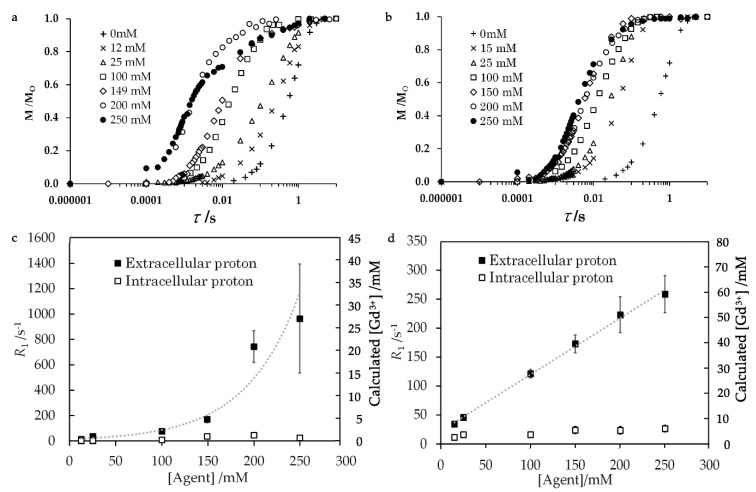
(**a**,**b**) Relaxation curves of the proton polarization for *T*_1_, (**c**,**d**) *T*_1_ relaxation rate and cellular Gd complex concentration estimated from the relaxivities of Gd complex. The samples were 40 μL of *E. coli-*glycerol mixtures to which (**a**,**c**) 10 μL of aqua Gd^3+^ or (**b**,**d**) Gd-DOTA solutions were added. The left vertical axes on panels *c* and *d* are scaled by the relaxivities of Gd complexes for the extracellular water only. Thus, the intracellular *T*_1_ relaxation rate is 73% of the values on these axes. The τ for the horizontal axis (**a**,**b**) gives the delay after the saturation pulse. Error bars were obtained from the deviation of experimental values from the best fit double exponential functions.

The water proton *T*_1_ relaxation rates increased with the concentration of added Gd solutions (Figure 2 and Table 1). The experimental details are given in Section 4.4. The extracellular and intracellular water resonances were distinguished by the difference in *R*_1_. The *T*_1_ relaxation curves in Figure 2a,b were fitted with multi-exponential buildup functions as described in Section 4.4 and Supplementary Materials S4 with Figure S2. The concentrations of Gd complex in the extracellular and intracellular parts in Figure 2c,d were calculated from the relaxivities of Gd complexes. These relaxivities were calculated from viscosities which were obtained from the experimental *E. coli* samples in the absence of the Gd complex. The relaxivities of aqua Gd^3+^ complex for the extracellular and intracellular solutions were 34.5 and 25.4 s^−1^ mM^−1^ and those of Gd-DOTA complex were 4.81 and 3.50 s^−1^ mM^−1^, respectively. These relaxivities were calculated using the Gd^3+^ proton distance of 3.2 Å as given in Section 4.1 and Supplementary Materials S5 and S6. Figure S3 and Table S1 show that the calculated relaxivity gave a Gd^3+^ concentration in a solution with accuracy of about 4%. Although the relaxivity of the aqua Gd^3+^ complex for extracellular water was only about 1.4-fold higher than that for intracellular water, the *T*_1_ relaxation rate of extracellular water was three times faster than the intracellular water *T*_1_ relaxation rate at every addition of Gd complex solution with different Gd concentrations. This result explains that the concentration of the extracellular aqua Gd^3+^ concentration was significantly higher than the intracellular concentration. The extracellular *T*_1_ relaxation rate non-linearly increased with the concentration of added aqua Gd^3+^ solution, as shown in Figure 2c.

**Figure 3:**
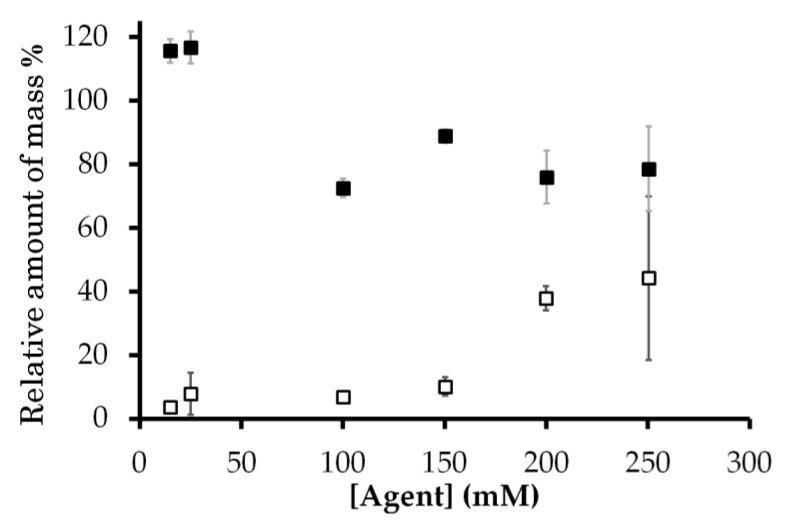
Amount of mass of Gd^3+^ ion in the sample calculated from *T*_1_ relaxation rate relative to the known amount of aqua Gd^3+^ (open square) and Gd-DOTA (closed square) added to the sample. The horizontal axis is the concentration of 10 μL Gd complex solutions added to the cell mixture. Error bars in Figure 3 were derived from those in Figure 2.

The amount of Gd^3+^ was calculated from the intracellular and extracellular proton relaxation rates *R*_1_. This amount relative to the total amount of Gd^3+^ added as solution with the known aqua Gd^3+^ and Gd-DOTA concentrations is shown in Figure 3. This figure shows that the cellular Gd amount obtained from the relaxivity was equal to or less than the Gd amount added to the samples, which confirms the validity of the Gd concentration obtained from the relaxivity. The relative Gd^3+^ amount was much lower than 100% only for aqua Gd^3+^. The reduction of this amount, the capability of Gd^3+^ for PRE, indicates that the exchangeable water molecules were prevented from accessing the unpaired electrons in Gd^3+^. Thus, the Gd amount gives the information for the interaction of Gd with cellular components.

In contrast to the *T*_1_ relaxation rate for aqua Gd^3+^, the *T*_1_ relaxation rate for the extracellular proton of the sample containing Gd-DOTA increased linearly in proportion to the added concentration (Figure 2d). The amount of Gd-DOTA recalculated from *T*_1_ relaxation rates relative to the known amount of Gd-DOTA was about 76–116% (Figure 3). Therefore, these *T*_1_ relaxation rates indicate that Gd-DOTA was stable and dissolved in the *E. coli* mixture.

### 2.3. High-Resolution Solid-State ^13^C NMR Spectra

**Figure 4:**
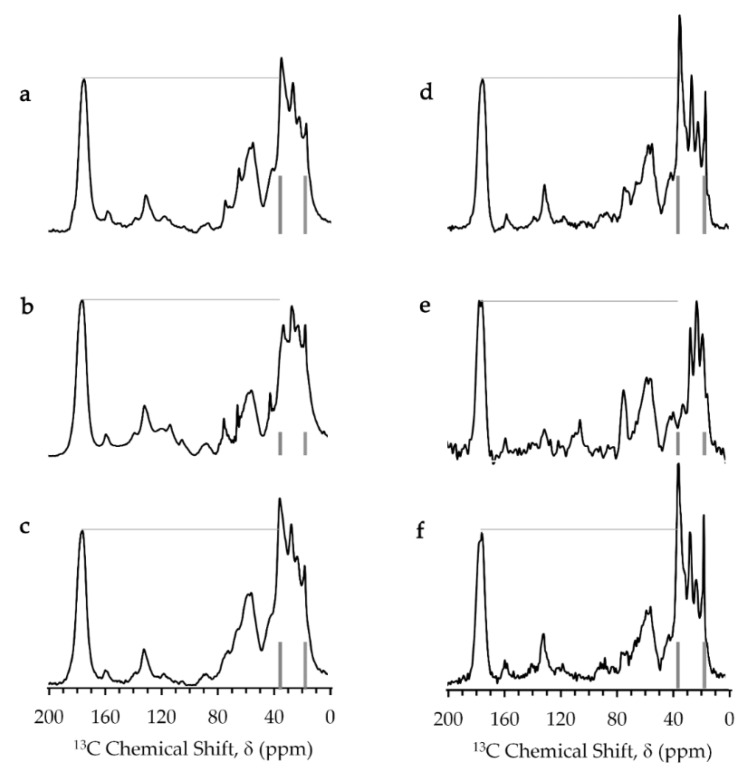
Solid-state ^13^C-NMR spectra obtained by CPMAS (cross polarization magic-angle spinning, right column) and single 90˚ pulse (left column) experiments for *E. coli* samples containing (**a**,**d**) NaCl, (**b**,**e**) aqua Gd^3+^ and (**c**,**f**) Gd-DOTA solutions. Carbonyl peak height and resonances for methyl carbon (16.8 ppm) and methylene carbon (35.5 ppm) of membrane lipids are marked by lines to guide the eye.

The single pulse and CPMAS (cross polarization magic-angle spinning) experiments give solid-state ^13^C-NMR spectra for all the static and mobile parts and those for only static parts of the *E. coli* cell samples, respectively. Experimental details are given in Section 4.5. The single pulse and CPMAS experiments provided similar spectra for the *E. coli* samples in absence of paramagnetic agents at a temperature of 218 K (Figure 4c,f). Therefore, all the cellular components were in a static state at this low experimental temperature.

The magic-angle spinning solid-state ^13^C-NMR spectra of the *E. coli* mixture in presence of Gd-DOTA complex were similar to those in absence of Gd complexes. However, the *E. coli* sample containing the aqua Gd^3+^ complex provided the spectra exhibiting an obvious reduction in height for the peak at 35.5 ppm which was assigned to the methylene carbon in the acyl chains of membrane lipids [35]. This resonance had the highest signal intensity in the spectra of the sample containing Gd-DOTA and the gadolinium-free sample because *E. coli* cells have a high lipid composition of 9% [31]. This strong peak was suppressed only in the spectra of the *E. coli* sample containing aqua Gd^3+^ due to transverse PRE effect of Gd^3+^. PRE gives information on the distance between the Gd^3+^ ions and cellular components. The reduction of this peak indicates that Gd^3+^ formed complexes with phospholipids in the cell membranes [36,37]. The paramagnetic fields of Gd^3+^ should reduce more than half of the signal of lipid methylene carbons which locate approximately within a radius of 1 nm of Gd^3+^ ions [38,39]. The peak of the methyl carbon of the lipid at 16.8 ppm [40,41,42] was not much suppressed by this PRE effect because methyl carbons were about 2 nm away from Gd^3+^ ions in the interface region of the bilayer membranes. The Gd^3+^ concentration in cytosol would not have been sufficient to suppress the ^13^C resonance of proteins whose carbonyl and C^α^ signals appeared at 175 and 60 ppm.

### 2.4. Viability of E. coli Cells and Magnetic Relxation during the Experiments

**Figure 5:**
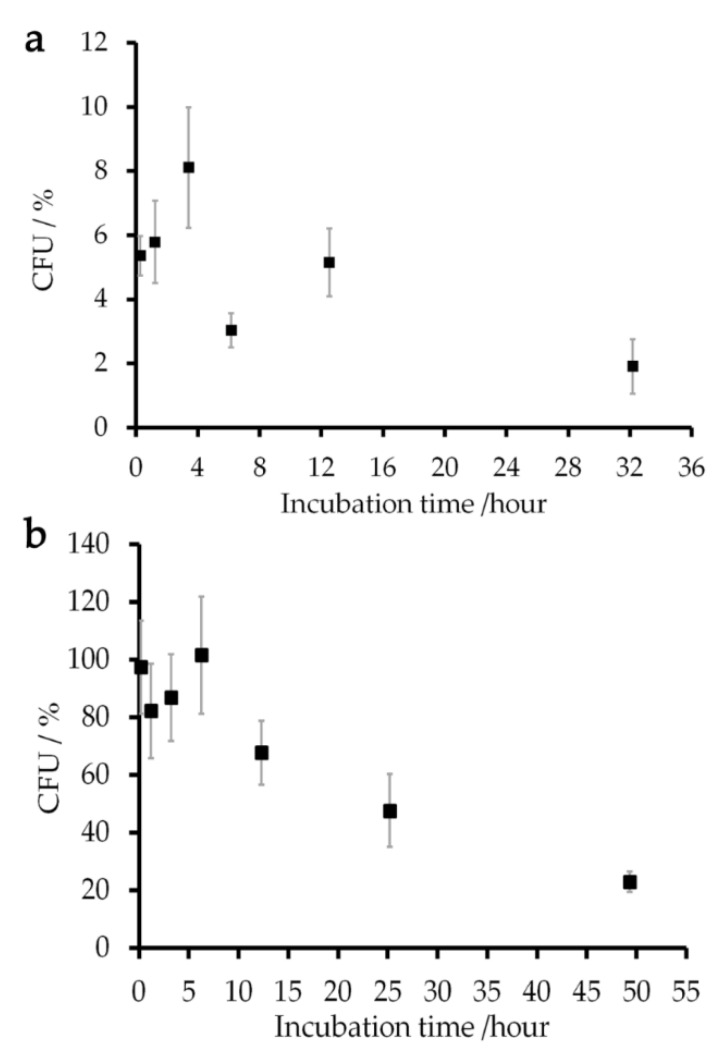
Percentages of CFU (colony-forming unit) of *E. coli* cells incubated in (**a**) 149 mM aqua Gd^3+^ and (**b**) 150 mM Gd-DOTA solutions relative to that of the sample incubated in a 150 mM NaCl solution are shown by closed squares. The error bars give the standard deviations of the results of two independent samples.

The viability of *E. coli* cells in the sample mixed with a 150 mM Gd-DOTA solution was examined by measuring their ability to form a new colony on an LB (Lysogeny Broth) plate or colony-forming unit (CFU) [43]. Since the samples mixed with 250 mM Gd solutions gave low reproducible results, the viability test was conducted using a concentration of Gd solutions at 150 mM which was close to the native salt concentration in cellular environment [44,45]. Experimental details are given in Section 4.6. The normalized CFU presented that around 90% of the cells were still alive in the Gd-DOTA solution for three hours (Figure 5). This value decreased exponentially with a time constant of 36 h. The NMR experiments for measuring *R*_1_ of the cells were completed within 40 min. Therefore, the experiments provided the *T*_1_ relaxation rates for the samples in which more than 90% of cells retained the colony-forming ability. The *E. coli* samples incubated in 149 mM aqua Gd^3+^ solution showed a CFU less than 10% after the incubation for 10 min. This figure also shows the cell viability during a series of *T*_1_ measurements which took almost ten hours.

**Figure 6:**
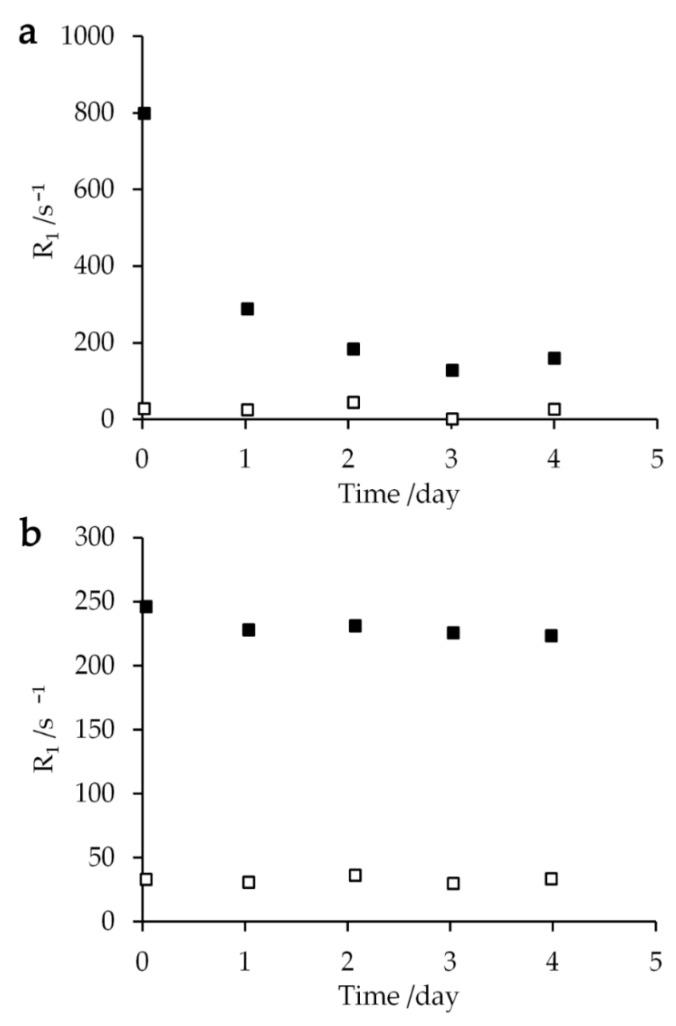
Extracellular (closed square) and intracellular (open square) relaxation rates *R*_1_ plotted against the time after mixing 10 μL of (**a**) 250 mM aqua Gd^3+^ or (**b**) 250 mM Gd-DOTA solutions with a 40 μL glycerol- *E. coli* sample at 273 K.

Relaxation rates *R*_1_ of the sample mixed with 250 mM aqua Gd^3+^ solution were measured repeatedly over several days in order to examine their dependence on the time elapsed after mixing the cell sample with the Gd solution. The extracellular relaxation rate *R*_1_ exponentially declined with a time constant of 1.09 ± 0.44 day (Figure 6a). Since the intracellular *T*_1_ relaxation rate and the amplitudes of extracellular and intracellular proton changed by less than 10%, the reduction of extracellular *T*_1_ relaxation rate should not occur due to the permeation of aqua Gd^3+^ complex into the cytoplasm or the cell lysis. Therefore, this decrease should reflect the complexation of Gd^3+^ ions with the cellular components. In contrast, the *E. coli* sample mixed with 250 mM Gd-DOTA solution showed that the intracellular and extracellular *T*_1_ relaxation rates did not change by more than 10% during the experiments for four days (Figure 6b). The slow reduction of the *T*_1_ relaxation rate can be interpreted as the initial part of an exponential decrease with a time constant of 41.7 ± 6.1 day. Thus, Gd-DOTA is better than aqua Gd^3+^ in that Gd-DOTA provides the stable PRE effect on the resonance of the cell samples for a longer period of time. We can obtain the information for the cell lysis and membrane permeability to Gd compounds as discussed in Section 3.2 and Section 3.3. The time constants longer than a day also show that Gd^3+^ and Gd-DOTA concentration-dependent NMR spectra in Figure 1 and *R*_1_ in Figure 2 were the same as those obtained immediately after the entry of Gd^3+^ and Gd-DOTA.

## 3. Discussion

### 3.1. Dependence of ^1^H-NMR Relaxation Rates R_1_ on Gd Complex Concentration

The relaxation rate *R*_1_ for the signal at around 4.7 ppm can be influenced by protons other than water protons such as methylene and methine protons in glycerol. However, those glycerol protons should have a small effect on the averaged *T*_1_ relaxation rate, less than by about 20%. This is because the *T*_1_ relaxation rates of methylene and methine protons were slower than that for water protons, e.g., *R*_1_ for CH_2_/CH and water protons were 66 and 200 s^−1^, respectively, in a water–glycerol mixture at the Gd-DOTA concentration of 12 mM. Moreover, the glycerol proton content relative to water protons in our cell sample was less than about 20%. The broadening of all the peaks in the spectra in Figure 2b,d indicates that the resonances of methylene and methine protons experienced PRE similarly to the water peak. The PRE effect on all protons also decreases the discrepancy between the *T*_1_ relaxation rate of the water proton and the averaged *T*_1_ relaxation rate. It is worthwhile to note that the *T*_1_ relaxation rate of the extracellular proton was about ten-fold faster than that of the intracellular proton at the addition of 150 mM Gd-DOTA solution. This *T*_1_ relaxation difference is much larger than the effect of methylene and methane protons on the measured *T*_1_ relaxation rate. Therefore, the difference in the *T*_1_ relaxation rate especially at high concentrations of Gd can be used for distinguishing between extra- and intracellular parts of the sample.

### 3.2. Interaction of Gd Complexes with the Cells and Cytotoxicity

**Figure 7:**
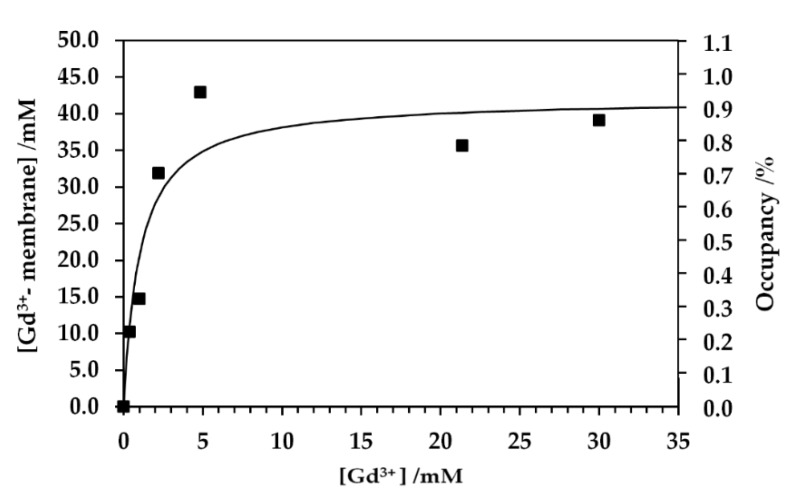
Concentration of Gd^3+^ ion binding with membrane of *E. coli* cells, [Gd^3+^- membrane], and the fractional occupancy of the binding site on cell membrane *Y* as a function of concentration of extracellular Gd^3+^ ion [Gd^3+^] are shown by closed squares. Experimental results were fitted to the function [Gd^3+^- membrane] = *S* ([Gd^3+^]/K_d_)/(1+([Gd^3+^]/K_d_)) where *S* is the concentration of total binding site *S* = [free cell membrane] + [Gd^3+^- membrane]. The percent occupancy is calculated from [Gd^3+^- membrane] × 100/*S*.

The weak PRE effect as observed in the *T*_1_ relaxation rate for the aqua Gd^3+^ titration NMR experiments of the cell sample was due to the binding of Gd^3+^ to cellular components, especially in anionic groups in lipids and macromolecules [36,37]. The Gd complex formation with cellular components sequesters the Gd^3+^ ion from bulk exchangeable water molecules and suppresses the PRE effect on the bulk water. The *T*_1_ relaxation rates in the aqua Gd^3+^ titration experiments were significantly low at the concentration for the addition of Gd^3+^ solution less than 150 mM (Figure 2c and Figure 3). This titration experiment provided a dissociation constant *K*_d_ of 1.0 mM for the Gd–cell component complex and the total binding site concentration of 42 mM (Figure 7). All the experimental data for *K*_d_ measurement were obtained from the *T*_1_ measurements given in Figure 2c,d and Figure 3. In this analysis, the number of the Gd–cell complex was obtained by subtracting the number of Gd^3+^ ions in the extracellular solution from the total number added to the sample. The Gd^3+^ ions should be transported to the periplasmic space through the porin channels in the outer membranes. Under the assumption that Gd^3+^ bound to the membranes in the cell surface and the periplasm, the amount of total binding sites calculated from the titrations was converted to the surface area of lipid membrane per binding Gd^3+^ ion of about 0.4 nm^2^. This amount was calculated using the number of the cells, the cell surface area and extracellular volume. The area of lipid membrane per binding Gd^3+^ ion of 0.4 nm^2^ is close to the area per lipid molecule of 0.6 nm^2^ [31,46]. Since only about a quarter of phospholipids in *E. coli* are known to have negative charges [47], Gd^3+^ ions would bind to not only anionic head groups of phospholipids [36,37] but also phosphates of neutral lipids and negatively charged biomolecules in the membranes and periplasm. The ^13^C-NMR spectra supported the interaction mode of Gd^3+^ with *E. coli* cells (Figure 4). The spectra show that the PRE effect of Gd^3+^ specifically suppressed the intensity of the lipid methylene ^13^CH_2_ peak by about 80%.

Note that the addition of 149 or 250 mM aqua Gd^3+^ solution to the cell sample caused cell aggregation and difficulty in pipetting the sample. Similar phenomena did not occur when Gd-DOTA solution was added to the cell sample. Such macroscopic difference in the cell aggregation is possibly related to the electric charge state altered by Gd^3+^ ions binding to the cell membranes.

The strong cytotoxicity of aqua Gd^3+^ as observed in the low CFU would be due to the binding of this ion to phospholipids which disrupts the interaction of the cell membranes with functional proteins by changing the electrostatic properties. Although the Gd^3+^ ions interacted mainly with anionic head groups of the membrane lipids, a part of the Gd^3+^ ions should have bound to proteins. Their binding to cellular proteins should alter the protein–ligand and protein–protein interactions by electrostatic forces. Such modification of biomolecular interactions should damage the cellular functions for metabolism and proliferation. Note that this toxicity did not directly correlate with the cell lysis because the *R*_1_ did not exhibit an increase in Gd^3+^ concentration in the intracellular part.

In contrast to aqua Gd^3+^, Gd-DOTA was stable in the samples, and its metal center did not bind strongly with components of *E. coli* cell. Therefore, Gd-DOTA could keep its Gd^3+^ ion accessible to water molecules. This stability caused Gd-DOTA to increase the *T*_1_ relaxation rate of water proton more efficiently than aqua Gd^3+^, although the number of water molecules in the coordination sphere of the Gd-DOTA complex was only one, while that of the aqua Gd^3+^ complex was eight.

The toxicities of Gd-DOTA and aqua Gd^3+^ complexes were also studied by other research groups. The toxicity of aqua Gd^3+^ complex is well known [14]. Similarly to our experimental results, Fuma et al. reported that the population of *E. coli* DH5α cells in culture medium suddenly decreased when the cell sample was exposed to 3 mM aqua Gd^3+^ solution [48]. Even though Gd-DOTA is a stable complex used as a contrast agent for MRI, an animal test showed that this complex accumulated in animal cells. Taupitz et al. found that the Gd-DOTA complex released its Gd^3+^ ion under an in vivo environment through transchelation with glycosaminoglycan [49].

On the basis of the data in our study, the stable Gd complex e.g., Gd-DOTA complex, is an appropriate paramagnetic agent for NMR spectroscopy of the living cells. A typical extracellular concentration of 40 mM did not damage *E. coli* cells. The concentration is close to the natural concentration of salt in *E. coli* cells [44,45]. Even this concentration is one order higher than the safety clinical dose for an MRI study and more than 80% of *E. coli* cells were alive for three hours under this condition. *E. coli* cells should be alive in our solution NMR experiments because the NMR experiment was finished within about one hour. It should be noted that the decrease of extracellular *T*_1_ relaxation rate of *E. coli* cells containing Gd-DOTA complex with time constant 41.7 days (Figure 6) is considerably slower than the death rate (Figure 5) of the cells with time constant 1.5 days. Therefore, the inactive cells in terms of proliferation should have an intact form in the membrane permeability to small molecules within a few days after the addition of Gd-DOTA.

### 3.3. Water Exchange Across the Cell Membranes and Its Effect on Water ^1^H T_1_ Relaxation

The *T*_1_ relaxation curves were fitted with a double exponential equation to analyze the *T*_1_ relaxation rate on the assumption that the water exchange between the intracellular and extracellular spaces was slower than the *T*_1_ relaxation rate. This assumption has been shown to explain the PRE effect on cellular water [50,51,52]. Without Gd complexes, *T*_1_ relaxation components with *R*_1_ = 0.9 and 1.5 s^−1^ were obtained. To distinguish the two *T*_1_ relaxation components, exchange rate *k*_ex_ should not be greater than 2 s^−1^ [50,51,52,53]. This rate corresponded with a relaxographic study of a yeast suspension at 298 K, which suggested a slow water exchange rate across the membrane of 1.49 s^−1^ [54]. Taking account of the temperature-dependence of *k*_ex_ [55], the *k*_ex_ of the cell sample at 273 K was lower than 1.49 s^−1^. Under the presence of Gd complexes, *R*_1_ >> 2 s^−1^ ≥ *k*_ex_, so that *k*_ex_ can be neglected particularly at high Gd concentrations. This negligence is confirmed by the observation of two *T*_1_ relaxation components differing by a factor of 10 in the *R*_1_ as shown in Figure 2c,d. The Gd complex concentrations were computed from the PRE effect obtained by subtracting *R*_1_ measured in the absence of Gd complexes from *R*_1_ in the presence of Gd complexes. Therefore, *k*_ex_ was suppressed in the calculation of the concentrations.

### 3.4. Cell Membrane Permeability to Gd Complexes

The stable and non-stable Gd complexes such as Gd-DOTA and aqua Gd^3+^ complexes are respectively known to penetrate into eukaryotic cells in a similar level [20,21,22]. Our study also showed the permeability of cell membranes of *E. coli* to those Gd complexes. The concentrations of Gd-DOTA and aqua Gd^3+^ were about 4 and 1 mM, respectively, in the cells irrespective of the concentrations in the extracellular space. The ratio of the extracellular to intracellular gadolinium complex concentrations increased with the extracellular concentration. Therefore, we can clearly distinguish between the extracellular and intracellular water resonances from the *T*_1_ relaxation time.

## 4. Materials and Methods

### 4.1. Data Analysis for the Longitudinal ^1^H Relaxation Rate R_1_ at High Magnetic Fields

We focus on effects of gadolinium paramagnetic agents on the longitudinal relaxation time *T*_1_ of water protons. In an aqueous solution containing a paramagnetic agent under high magnetic fields, the dipole–dipole interactions between unpaired electron and proton spins mainly causes the *T*_1_ relaxation [56,57]. The relaxation time *T*_1_*_m_* due to the dipolar coupling with the unpaired electron spin depends on the total correlation time $\tau_{c}$ and the proton–electron distance *r* as expressed by the Solomon–Bloembergen–Morgan equation [58,59,60]:

(1) $$\frac{1}{T_{1m}}=\frac{2}{15}{\left(\frac{\mu_{B}\gamma_{H}\gamma_{e}\hbar }{r^{3}}\right)}^{2}S(S+1)\left\{\frac{{3\tau }_{c}}{{1+\omega }_{H}^{2}\tau_{c}^{2}}+\frac{{7\tau }_{c}}{{1+4\omega }_{e}^{2}\tau_{c}^{2}}\right\}\;$$

(2) $$\frac{1}{\tau_{c}}=\frac{1}{T_{1e}}+\frac{1}{\tau_{r}}+\frac{1}{\tau_{m}}$$

where γ*_i_* and *ω_i_* are, respectively, the gyromagnetic ratio and Larmor frequency for electron *i* = *e* and proton *i* = H. *S* is the spin quantum number of the paramagnetic ion; *τ_r_* and *T*_1*e*_ are rotational correlation time and electron spin relaxation time of the paramagnetic complex, respectively; and *τ_m_* is the water residency time during which a water molecule resides in the coordination sphere of the complex. The relaxivity *R*_1_*_p_*, the *T*_1_ relaxation rate of the sample enhanced by 1 unit in the concentration of the paramagnetic agent, can be calculated as a function of *T*_1_*_m_* and *τ_m_* [58,59,60,61]:

(3) $${\;R}_{1p}=\frac{q}{{[H}_{2}O]}\left(\frac{1}{T_{1m}{+\tau }_{m}}\right)$$

where *q* is the number of water molecules in the inner coordination sphere of the complex. This relaxivity can also be expressed with the concentration of the paramagnetic ion as

(4) $${\;R}_{1p}=\frac{R_{1}^{\prime }{\;-\;R}_{1}^{0}\;}{\left[Paramagnetic\;agent\right]}$$

where $R_{1}^{\prime }$ and $R_{1}^{0}$ are the relaxation rates of the samples with and without the paramagnetic agent, respectively.

Under magnetic fields higher than 3 T, the effect of *T*_1*e*_ on the *T*_1_ relaxation rate of the water proton is known to be neglected [62,63]. At 298 K, *T*_1*e*_ calculated from parameters reported [45,64,65] is approximately 1.35 × 10^−7^ and 2.01 × 10^−6^ s for aqua Gd^3+^ and Gd-DOTA complexes respectively. The time constant for the rotational diffusion is much shorter than that for the exchange between the inner sphere and the outer sphere of gadolinium complexes as ($\tau_{r}$, $\tau_{m}$) = (2.9 × 10^−11^ s, 1.2 × 10^−9^ s) and (9.0 × 10^−11^ s, 2.0 × 10^−7^ s) at 298 K for aqua Gd^3+^ and Gd-DOTA complexes, respectively [65,66]. Because $\tau_{r}$ << $\tau_{m}$<< *T*_1*e*_, Equation (2) is expressed as 1/$\tau_{c}$ ≈ 1/$\tau_{r}$ by neglecting $\tau_{m}$ and *T*_1*e*_. The relaxivity of this solution in Equation (3) can be approximated by replacing *T*_1_*_m_* + $\tau_{m}$ with *T*_1_*_m_*.

### 4.2. Aqua Gd^3+^ and Gd-DOTA Solutions

The solutions with aqua Gd^3+^ and Gd-DOTA complexes were prepared by dissolving GdCl_3_·6H_2_O (Wako Pure Chemical Ind., Osaka, Japan) and Gd-DOTA (BOC Sci., Shirley, NY, USA) in Milli-Q water (Merckmillipore, Darmstadt, Hessen, Germany), respectively.

### 4.3. E. coli Samples

BL21 Star^TM^ (DE3) *E. coli* cells were grown in 120 mL of 20 g/l LB medium containing 50 μg/mL ampicilin at 37 °C until OD_600_ = 0.7–0.8. The culture was harvested by centrifuging at 2000 g for 20 min at 4 °C. The suspension was then washed with 0.14 M NaCl solution and centrifuged at 3300 g for 15 min at 4 °C. The suspension was washed again with 0.14 M NaCl solution and centrifuged at 3300 g for 5 min at 4 °C. The suspension was afterward mixed with 99.0% (mass/mass) glycerol (Wako Pure Chemical Ind., Osaka, Japan) so that the glycerol concentration was 20% *w*/*w* before adding aqua Gd^3+^ and Gd-DOTA solutions. *Escherichia* *coli* cells uniformly labeled with ^13^C used for ^13^C-NMR experiments were prepared similarly, but the LB medium was replaced with M9 medium containing [U-^13^C] glucose, ^14^NH_4_Cl, nucleosides and 50 μg/mL ampicilin. Solutions with 10 μL of aqua Gd^3+^ at 0, 12, 25, 100, 149, 200, 250 mM and those of Gd-DOTA at 15, 25, 100, 150, 200, 250 mM were added to 40 μL of glycerol-*E. coli* mixtures 20 min before NMR experiments.

### 4.4. Relaxation Measurement of the ^1^H Longitudinal Magnetization

A 20 μL glycerol-*E.coli* sample containing Gd was transferred into a glass tube (NORELL, Morganton, NC, USA) cut to fit in a magic-angle spinning (MAS) solid-state NMR probe for a 4.0 mm rotor. This tube was capped with Parafilm. The *T*_1_ relaxation of proton magnetization was measured with a saturation pulse train followed by an excitation pulse at 273 K on an ECAII NMR spectrometer (JEOL Ltd., Akishima, Tokyo, Japan) equipped with a Balun probe (Varian, Palo Alto, LA, USA) at the ^1^H resonance frequency of 700 MHz. The RF amplitude for the pulses was 50 kHz, and the relaxation delay was 7s. The measurement was finished within 40 min after mixing the cell sample with a Gd solution. The samples were kept at 277 K in a refrigerator for the measurement of *R*_1_, i.e., 1/*T*_1_, as a function of the elapsed time after the sample preparation.

The buildup curves of the integral area of signal intensity were fitted to the double exponential relaxation equation for *T*_1_ as

(5) $$M\;(t)=M_{o,\mathrm{ex}}{\;(1-\;\exp\;(-t/T}_{1,\mathrm{ex}}{))+M}_{o,\mathrm{in}}{(1-\;\exp\;(-t/T}_{1,\mathrm{in}}))+c$$

where the subscripts ‘ex’ and ‘in’ stand for extracellular and intracellular parts, respectively.

### 4.5. Solid-State ^13^C-NMR Experiments

Three samples different in composition were prepared by mixing 80 μL of ^13^C-labeled *E.coli*-glycerol solutions with 20 μL of 140 mM NaCl, 149 mM aqua Gd^3+^ or 150 mM Gd-DOTA solutions. The samples were transferred into a 4.0 mm MAS rotor and frozen rapidly in liquid nitrogen. The ^13^C-NMR signals at the resonance frequency 125 MHz were acquired under ^1^H decoupling via a single ^13^C 90˚ pulse or a ^1^H 90˚ pulse followed by a cross polarization (CP) period with a contact time of 2 ms. These experiments were performed on the NMR spectrometer equipped with a T3 triple-resonance probe at 218 K and the relaxation delay of 7 s under MAS at the frequency of 12 kHz.

### 4.6. Viability of E. coli Cells

Samples with 80 μL of living *E. coli*-glycerol were mixed with 20 μL of 150 mM Gd-DOTA or 149 mM aqua Gd^3+^ solutions. The samples were kept at 0 °C in an ice bath before serial dilution. After incubation, the *E. coli* samples were diluted in 140 mM NaCl solution. The diluted *E. coli* samples were then cultured on an LB agar plate at 37 °C. The number of colonies were counted. A 40 μL sample of living *E. coli*-glycerol was also mixed with 10 μL of 150 mM NaCl solution as a control. This sample was diluted rapidly after mixing and cultured on an LB plate. The number of colonies on an LB plate of the sample containing Gd was normalized by the number of colonies of the control *E. coli* sample.

## 5. Conclusions

Gadolinium complexes enhanced the longitudinal relaxation rate *R*_1_ of extracellular water protons, selectively owing to the low permeability of cell membranes to the agents and the low water exchange rate across the membranes. The *R*_1_ of the extracellular part was more than ten times faster than the intracellular *R*_1_, which allowed discrimination between extra- and intracellular parts. The ^1^H-NMR relaxation for *T*_1_ provided the concentrations of gadolinium complexes in the inside and outside of *E. coli* cells, which revealed the cell membrane permeability to Gd complexes. The PRE effect in solid-state ^13^C-NMR indicated that aqua Gd^3+^ bound to lipid membranes strongly but Gd-DOTA did not. This binding with a dissociation constant of 1 mM should be the cause for the cytotoxicity of aqua Gd^3+^ stronger than that of Gd-DOTA. The results of the molecular interaction studies confirmed that Gd-DOTA was more suitable than aqua Gd^3+^ as an *R*_1_ contrast agent for cellular localization study by NMR spectroscopy. It had lower cytotoxicity even at higher concentrations around 30 mM which enhanced *R*_1_ for the extracellular space selectively, and it exhibited a strong PRE effect through the exchange of ligand water with bulk water by staying in the solution phase for a period longer than four days.

## Figures and Tables

**Table 1:** Intracellular and extracellular *T*_1_ relaxation rates and amplitudes of 40 μL of glycerol*-E. coli*- sample to which 10 μL Gd-DOTA or aqua gadolinium solution was added.

Agent	[Agent]	Intracellular Solution		Extracellular Solution	
	(mM)	*R*_1_ (s^−1^)	Amplitude (%)	*R*_1_ (s^−1^)	Amplitude (%)
	0	1.5 ± 0.3	32 ± 25	0.89 ± 0.03	68 ± 25
	15	12 ± 0	84 ± 1	34 ± 1	16 ± 1
	25	16 ± 0	63 ± 5	45 ± 1	37 ± 5
Gd-DOTA	100	16 ± 3	44 ± 0	121 ± 7	56 ± 0
	150	34 ± 6	32 ± 4	182 ± 15	68 ± 4
	200	24 ± 7	35 ± 3	223 ± 30	65 ± 3
	250	26 ± 6	27 ± 1	259 ± 32	73 ± 1
	12	1.7 ± 0.2	75 ± 3	14 ± 3	25 ± 3
	25	3.6 ± 2.3	65 ± 10	36 ± 21	35 ± 10
[Gd(H_2_O)_8_]^3+^	100	10 ± 1	38 ± 3	77 ± 2	62 ± 3
	149	37 ± 11	44 ± 16	170 ± 26	55 ± 16
	200	47 ± 21	26 ± 6	744 ± 124	74 ± 6
	250	25 ± 7	25 ± 3	965 ± 429	75 ± 3

## Supplementary Materials

Supplementary materials can be found at https://www.mdpi.com/1422-0067/21/11/4042/s1. Supplementary Material S1. Amount of glycerol and water in *E. coli* samples; Supplementary Material S2. T1 of water proton in absence of paramagnetic agent; Supplementary Material S3. T1 of water proton in cell samples; Supplementary Material S4. Relaxation buildup curves fitted with multi-exponential functions; Supplementary Material S5. Validation of parameters for estimating the relaxivity; Supplementary Material S6. Relaxivity of Gd solutions in *E. coli* cell samples. References [67,68,69,70,71,72,73,74] are cited in the supplementary materials.

## Abbreviations

Gd-DOTA	Gadolinium-1,4,7,10-tetraazacyclododecane-1,4,7,10-tetraacete complex
NMR	Nuclear Magnetic Resonance
MRI	Magnetic Resonance Imaging
*R*_1_	Longitudinal relaxation rate
*T*_1_	Longitudinal relaxation time
*R*_1_ *_p_*	Longitudinal relaxivity
PRE	Paramagnetic Relaxation Enhancement
CPMAS	Cross Polarization Magic-Angle Spinning
CFU	Colony-forming unit
