# Supplementary material for: Gadolinium Complexes as Contrast Agent for Cellular NMR Spectroscopy
Source: Int J Mol Sci. 2020 Jun 5;21(11):4042. doi: 10.3390/ijms21114042 (PMC7312942; doi:10.3390/ijms21114042)
Supplement: Supplementary file 1 [file ijms-21-04042-s001.pdf]

Supplementary Materials

Gadolinium complexes as contrast agent for cellular NMR spectroscopy

Nat Sakol [1], Ayako Egawa [1] and Toshimichi Fujiwara [1,*]

[1] Institute for Protein Research, Osaka University, 3-2 Yamadaoka, Suita, Osaka 565-0871, Japan
* Correspondence: tfjwr@protein.osaka-u.ac.jp

S1. Amount of glycerol and water in *E. coli* samples

The *E. coli* sample was prepared as the method reported in the main text. The dried weight of the *E. coli* sample was measured from the sample lyophilized overnight. This experiment showed the remaining part of the sample was 10 ± 1 % of the sample mass. The intracellular water was approximated from this dried weight on the basis that 70% of the cellular weight was mass of water [32]. The extracellular water was calculated by subtracting the mass of intracellular water from the total mass of water in the sample. This method revealed that the extracellular and intracellular water weights in the *E. coli* sample were 68 ± 2% and 22 ± 2% of the sample weight, respectively. These amounts were used for estimating the amount of glycerol and water in the sample after adding glycerol and gadolinium solutions into *E. coli* cells. For example, *E. coli* sample of 80.00 mg consists of extracellular water of 53.33 mg and intracellular water of 18.67 mg. This sample then mixed with 20.00 mg of 99.0 % m/m glycerol. Given that the density of *E. coli* cell and glycerol was 1.11 and 1.26 g/ml [70,71], respectively, the total density of glycerol-*E. coli* mixture was 1.05 g/ml, and the compositions of glycerol, extracellular water, and intracellular water were 16.5, 56.4 and 19.7% of the sample volume, respectively. Then, NaCl or Gd solutions were added to 40.00 μl of this mixture. This 40.00 μl of *E. coli*-glycerol mixture contained 7.86 μl of intracellular water, 22.5 μl of extracellular water and 6.62 μl of glycerol. 10 μl of NaCl solution thus increased the fraction of extracellular water to 65.1% of the sample volume or 69.2% of cellular liquid. This changed the concentration of glycerol to 13.2% of sample volume or 14.1 % of liquid volume in the sample. On the condition that glycerol and NaCl solution did not penetrate into the cell, the concentration of glycerol in the extracellular part of the cell was 16.9% v/v. On the other hand, if all NaCl solution passed into the cell, the concentration of glycerol in extracellular part was 22.7% v/v.

S2. T_1 of water proton in absence of paramagnetic agent

The relaxation time of proton in pure water without paramagnetic agent can be calculated as

$$\frac{1}{T_1} = C\left\{\frac{2\tau_r}{1+4\omega_H^2\tau_r^2} + \frac{\tau_r}{1+\omega_H^2\tau_r^2}\right\} \tag{S1}$$

where ω_H is Larmor frequency of ^{1}H. The constant C was empirically estimated by fitting relaxation time reported by Hindman *et al* [67] to equation S1. The correlation time τ_r was calculated by the function:

$$\tau_r = \frac{4\pi A^3 \eta}{3k\mathrm{T}} \tag{S2}$$

where A is a radius of molecule of 1.4 Å and η is viscosity [68]. The viscosity was calculated from the equation of Fogel'son *et al* [69] as

$$\eta = \eta_0 e^{\frac{E}{k(\mathrm{T}+\mathrm{T}_0)}} \tag{S3}$$

where E = 4.7428 kJ mol^{-1}, τ_r = 2.4152 x10^{-5} Pa s and T_0 = –139.86 K.

This calculation gave the constant C of 3.69 × 10^{10} s^{-2}. We used this constant for computing the temperature dependent T_1 (Figure S1). Figure S1 reveals the good agreement between the calculated and experimental T_1 at proton resonance frequency of 500 MHz especially at temperature lower than 300 K where the celluar NMR experiments were performed. This agreement shows the validity of our theoretical framework for the analysis of the T_1 relaxation. The predicted values were slightly longer than the experimental results, which would be due to paramagnetic O_2 molecules in the experimental water.

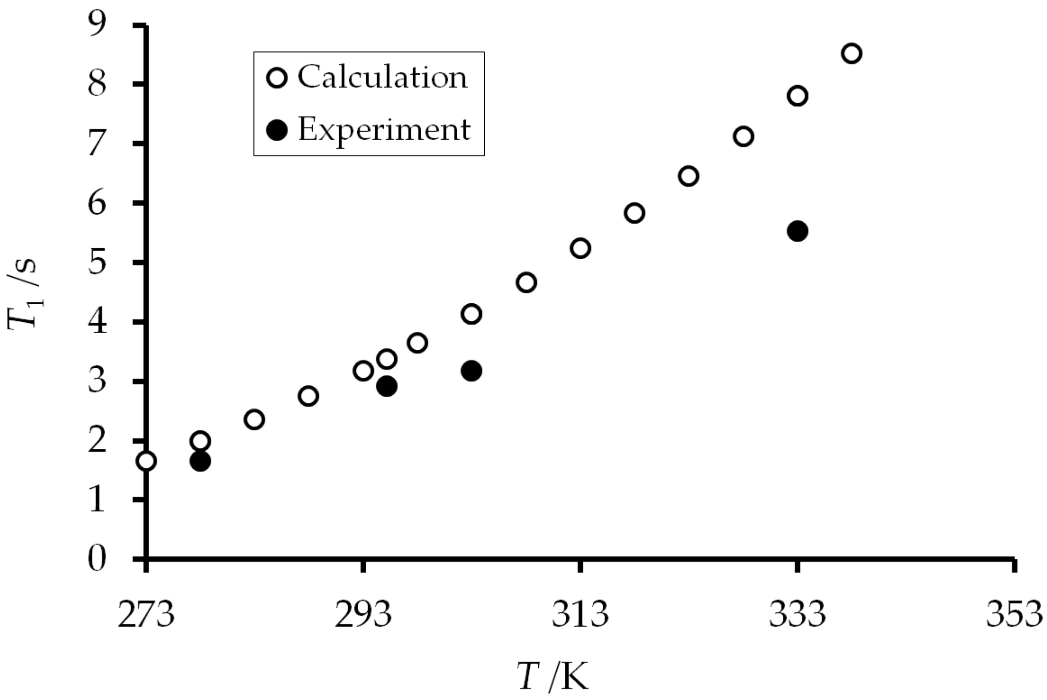

Figure S1: Theoretical T_1 calculated from the Bloembergen-Purcell-Pound equation (open circle) and experimental one (closed circle) plotted against temperature. The T_1 values were measured by the saturation recovery method as described in section 4.4 in the main manuscript.

S3. T_1 of water proton in cell samples

The experimental proton relaxation rates in gadolinium-free *E. coli* samples were 0.887 ± 0.035 and 1.51 ± 0.31 s^{-1} for extracellular and intracellular water, respectively, at the ^{1}H resonance frequency of 700 MHz and temperature of 273 K. These relaxation rates were used to compute viscosity and rotational correlation time from Equation S1. Given the constant C = 3.39 × 10^{-10} s^{-2} as in section S1, the extracellular relaxation rate of 0.887 s^{-1} revealed the viscosity of 2.64 mPa.s and τ_r = 8.06 ps.

The viscosity and rotational correlation time of intracellular water were also calculated based on Persson and Halle model [33,34]. According to this model, the intracellular water surrounding macromolecules consists of the first hydration layer water and the bulk water of 15% and 85%, respectively. Therefore, the relaxation rate of the intracellular water is calculated as the sum of the relaxation rates of the first hydration sphere water and the bulk water as:

$$R_{1,\text{intra}} = 0.15\, R_{1,\text{hyd}} + 0.85\, R_{1,\text{bulk}} \qquad \text{(S4)}$$

where subscripts 'intra', 'hyd' and 'bulk' stand for the intracellular, first hydration layer water, and bulk respectively.

The correlation time of the bulk water is 15.6 time shorter than that of the first hydration water in the model. By using Equation (S4),

$$\frac{1}{T_1} = 0.15C\left\{\frac{15.6\tau_{r,\text{bulk}}}{1+\omega^2(15.6\tau_{\text{bulk}})^2}+\frac{2\times 15.6\tau_{\text{bulk}}}{1+4\omega^2(15.6\tau_{\text{bulk}})^2}\right\} + 0.85C\left\{\frac{\tau_{r,\text{bulk}}}{1+\omega^2\tau_{r,\text{bulk}}^2}+\frac{2\tau_{\text{bulk}}}{1+4\omega^2\tau_{\text{bulk}}^2}\right\} \qquad \text{(S5)}$$

Equation (S5) provided $\tau_{r,\text{bulk}}$ = 5.36 ps and $\tau_{r,\text{hyd}}$ = 83.6 ps (= 15.6 x $\tau_{r,\text{bulk}}$). Thus, the viscosity was 27.4 and 1.76 mPa.s for the first hydration water and bulk water, respectively. The correlation time of bulk water at 273 K evaluated from equations in section S1 was 5.26 ps, which is close to the value calculated from relaxation time of the intracellular water.

S4. Relaxation buildup curves fitted with multi-exponential functions

The experimental relaxation times of NMR signal were analyzed by fitting buildup curves with double exponential relaxation equations. The experimental buildup curves agree with the double exponential relaxation function better than the single exponential function as shown in Figure S2. The components of the double exponential fitting curves were then interpreted as extracellular and intracellular relaxation curves based on the increase of relaxation rates.

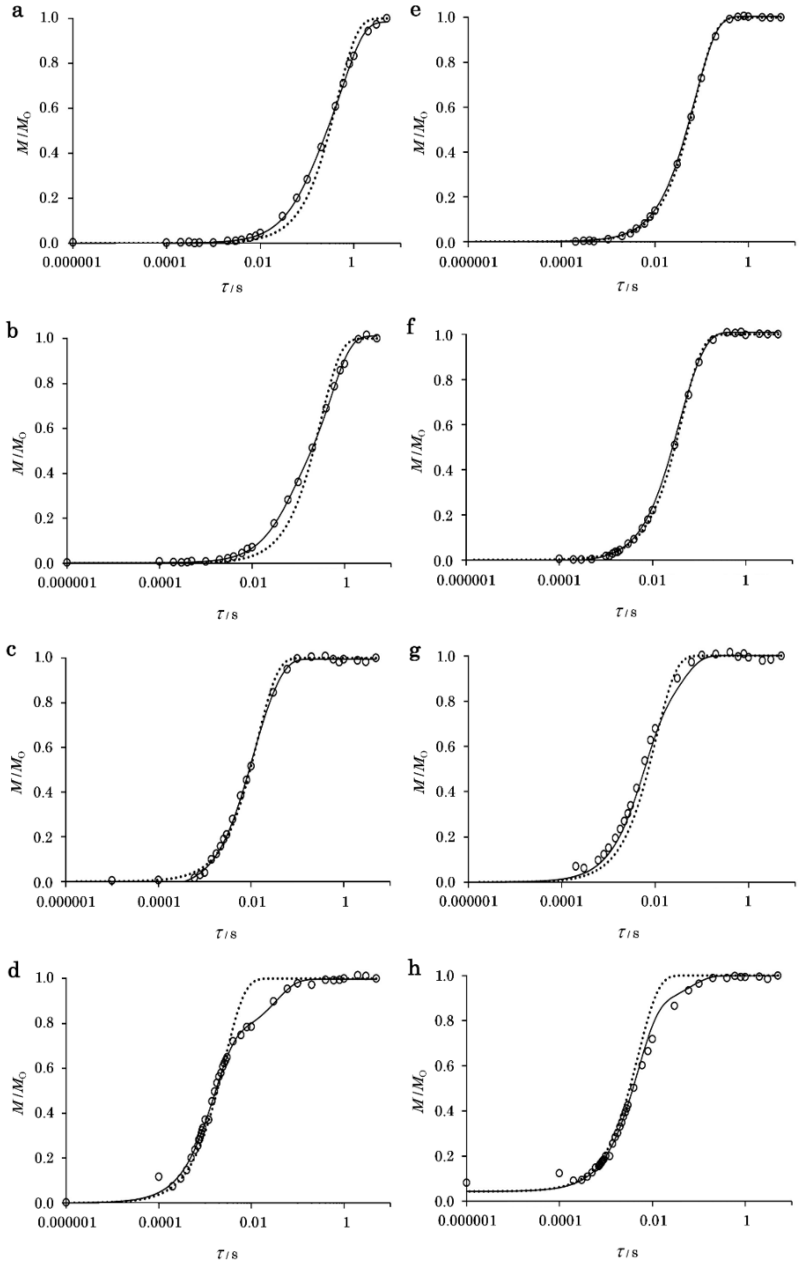

Figure S2: Experimental buildup curves of the mixture of 40 μl glycerol-*E. coli* and 10 μl aqua Gd^{3+} solutions at 12 (a), 25 (b) 149 (c) and 250 mM (d), and those of the mixture of 40 μl glycerol-*E. coli* and 10 μl of Gd-DOTA solutions at 15 (e), 25 (f), 150 (g), and 250 mM (h). The experimental buildup curves were fitted with the double exponential relaxation curves (solid lines) and single exponential relaxation curves (dotted lines). The τ for the horizontal axis gives the delay after the saturation pulse.

S5. Validation of parameters for estimating the relaxivity

The relaxivities of gadolinium agent were calculated from Equations 1-3 in the main text using parameters and compared with the experimental data of aqua Gd^{3+} solution. Given r = 3.2 Å [72], the correlation time τ_r was calculated from the radius of aqua Gd^{3+} complex of 3.87 Å [73]. Our experimental results (Figure S3) gave the relaxivity of aqua Gd^{3+} solution, 11.4 ± 0.2 s^{-1} mM^{-1} under $\omega_H/2\pi$ = 500 MHz and at 295 K. This value is close to the predicted relaxivity of 11.9 s^{-1} mM^{-1} (Table S1). The experimental relaxivity was calculated from Equation (4). The agreement between the predicted and experimental relaxivity shows that Equations 1-4 are applicable to calculating our experimental results.

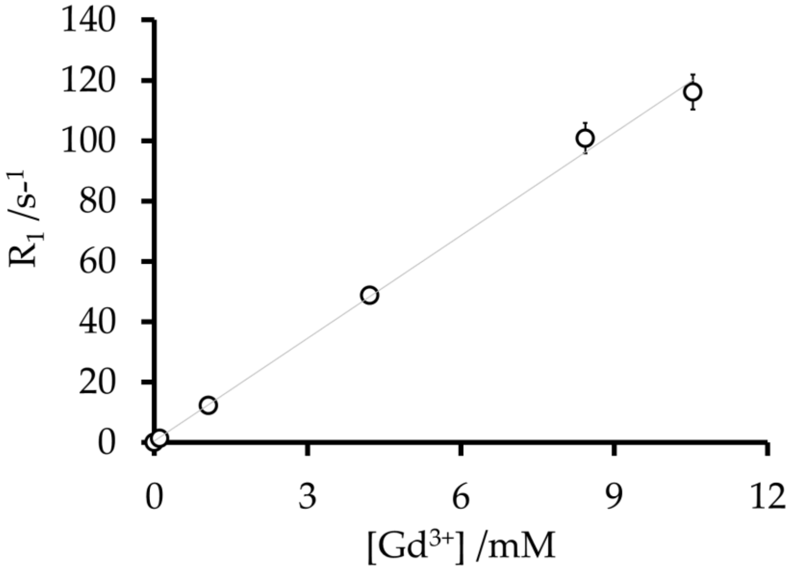

Figure S3: Proton relaxation rate of aqua Gd^{3+} solution plotted against the concentration giving the relaxivity of 11.4 ± 0.2s^{-1} mM^{-1}.

Table S1: Relaxivity (R_{1p}) of aqua Gd^{3+} complex calculated from Equations 1-3 in the main text

T (K)	η (Pa s)	τ_r (ns)	$\frac{3\tau_r}{1+\omega_H^2\tau_r^2}+\frac{7\tau_r}{1+4\omega_S^2\tau_r^2}$ (10^{-10})	T_{1m} (μs)	R_{1p} (s^{-1} mM^{-1})
278	0.001501	94.94	2.849	7.274	19.797
283	0.001299	80.73	2.426	8.541	16.861
288	0.001136	69.35	2.087	9.927	14.506
293	0.001002	60.11	1.812	11.432	12.596
**295**	**0.000955**	**56.90**	**1.717**	**12.067**	**11.933**
298	0.00089	52.54	1.587	13.054	11.031
303	0.000797	46.26	1.401	14.789	9.737
308	0.000718	41.02	1.246	16.634	8.657
313	0.000651	36.59	1.115	18.583	7.749

S6. Relaxivity of Gd solutions in *E. coi* cell samples

The relaxivity of aqua Gd^{3+} complex with eight coordinated water molecules was computed from Equations 1-3. The correlation time of this complex was calculated using the viscosity in section 1 and complex radius of 3.87 Å [72]. This gave a relaxivity of 34.5 s^{-1} mM^{-1} in extracellular part of the sample. The relaxivity in the intracellular solution was also computed. The relaxivity in the bulk and the first hydration sphere layer of intracellular solution were averaged as Equation S4 and gave the averaged relaxivity in the intracellular solution of 25.4 s^{-1} mM^{-1}.

The relaxivities of Gd-DOTA complex in extracellular and intracellular solution were also approximated by the above method using complex radius of 4.0 Å [74]. This gave the relaxivities of this complex in the extracellular and intracellular solution of 4.81 and 3.50 s^{-1} mM^{-1}, respectively.
